# Cognition in zero gravity: Effects of non-terrestrial gravity on human behaviour

**DOI:** 10.1177/17470218221113935

**Published:** 2022-08-06

**Authors:** Iqra Arshad, Elisa Raffaella Ferré

**Affiliations:** 1 Department of Psychology, Royal Holloway, University of London, Egham, UK; 2 Department of Psychological Sciences, Birkbeck, University of London, London, UK

**Keywords:** Spaceflight, gravity, sensorimotor functioning, cognition, social and affective processing, extreme environments

## Abstract

As humanity prepares for deep space exploration, understanding the impact of spaceflight on bodily physiology is critical. While the effects of non-terrestrial gravity on the body are well established, little is known about its impact on human behaviour and cognition. Astronauts often describe dramatic alterations in sensorimotor functioning, including orientation, postural control, and balance. Changes in cognitive functioning as well as in socio-affective processing have also been observed. Strikingly, no comprehensive theoretical model exists to outline the impact of non-terrestrial gravity on behaviour. Here, we have reviewed the key literature across the last 10 years and explored the impact of non-terrestrial gravity across three key functional domains: *sensorimotor functioning, cognition, and socio-affective processing*. We have proposed and preliminary validated a neurocognitive model to account for the effects of non-terrestrial gravity in these domains. Understanding the impact of non-terrestrial gravity on human behaviour has never been timelier and it will help mitigate against risks in both commercial and non-commercial spaceflight.

## Introduction

Human missions to Mars and the Moon along with commercial ventures for space travel are fast becoming a reality. As humanity prepares for a *new space exploration age*, understanding the impact of spaceflight on the human body and brain has never been timelier. Space is an extremely hostile environment. Astronauts face both physical and mental challenges; ionising radiation, absence of circadian rhythms and confinement and isolation, which are combined with prolonged exposure to non-terrestrial gravities. While the effects of non-terrestrial gravities on human bodily physiology, such as the musculoskeletal (Lang et al., 2017) and cardiovascular (Aubert et al., 2016; Tanaka et al., 2017) systems, are well-documented, relatively little is known about the impact of altered gravity on the human brain and behaviour. That is, most current scientific work on the effects of non-terrestrial gravity on cognition has largely been driven by isolated observations of behavioural alterations. As a result, no comprehensive view of the effects of both actual and simulated non-terrestrial gravity on neurocognitive functioning has been developed. Notable previous work has outlined the impact of spaceflight on the human brain and behaviour; however, a clinical ageing population was used as a point of comparison (Hupfeld et al., 2021). Here, we critically review key findings across the last 10 years of space research to provide a framework of *cognition in zero gravity* exploring findings in healthy individuals.

Since the beginning of time, all living organisms have evolved under a terrestrial gravitational acceleration of 9.81 m/s^2^, also called 1*g*. It is hard to imagine a more fundamental and ubiquitous aspect of life on Earth than gravity. On Earth, sophisticated organs in the inner ear — the vestibular otoliths — detect gravitational acceleration. When the head moves with respect to gravity, the vestibular otoliths shift with the direction of gravitational acceleration, moving the hair cell receptors and signalling to the brain the position of the head relative to the gravitational vector. Vestibular signals are integrated with sensory inputs from vision, proprioception, and viscera, as well as semantic knowledge and past experiences, to form an *1g Internal Model of Gravity* (Jörges & López-Moliner, 2017; Lackner & DiZio, 2005; Lacquaniti et al., 2015; Zago & Lacquaniti, 2005a, 2005b). The physical constraints of Earth’s gravity are therefore internalised in the human brain, and the 1*g* Internal Model of Gravity exploits them to form accurate perceptions of the external environment (Gallagher et al., 2021). Perhaps not surprisingly, we are exceptionally well-adapted to the acceleration of terrestrial gravity (Barra et al., 2010; Green et al., 2005; Indovina et al., 2005). Accordingly, random accelerations are hardly perceived at all (Brenner et al., 2016; Werkhoven et al., 1992), falling objects are expected to accelerate even when their velocity is constant (Zago et al., 2004), and observers generally misremember the location of moving objects in space, displacing them as if they were under the influence of terrestrial gravity (de Sá Teixeira et al., 2013). People can also catch objects accelerating downwards with little to no effort, even when parts of the object’s trajectory are occluded and no cues about position and velocity are given (Lacquaniti & Maioli, 1989; Monache et al., 2019; Zago et al., 2005). Our lifelong experience with gravity makes the 1*g* Internal Model of Gravity highly reliable and optimal for terrestrial environments.

Since the first space missions, however, it has been clear that adjusting to non-terrestrial gravities takes time and effort. The absence of gravity during spaceflight leads to the unloading of vestibular otoliths such that they no longer become stimulated as they would on Earth by changes in the head’s spatial orientation. Animal models have suggested that this unloading causes both structural and functional changes in the vestibular organs. For instance, an increase in the mass of otoconia (Boyle & Varelas, 2021) and a reduction in the synapse densities of the hair cells have been observed following microgravity exposure (Sultemeier et al., 2017). These alterations in the otoliths have been shown to lead to changes in vestibular central processing, including an increase in the sensitivity of vestibular pathways (Cullen, 2019). Consequently, sensory conflicts may arise between the 1*g* Internal Model of Gravity and the unusual gravitational information signalled by the vestibular otoliths. For example, when an astronaut walks on the lunar surface, the 1*g* Internal Model of Gravity is no longer optimal, and the brain must rely on online lunar 0.16*g* signals transmitted by the vestibular otoliths to successfully guide behaviour. Accordingly, astronaut reports have shown changes in the central nervous system during and after spaceflight in the form of *neurovestibular problems* (Van Ombergen, Laureys, et al., 2017a, 2019). Importantly, astronauts are also subjected to several stressors including workload, confinement and isolation, circadian rhythm changes, dietary changes such as insufficient food and nutrition, communication delays, distance from Earth, and teamwork stressors (Kanas & Manzey, 2008) along with physical challenges (Patel et al., 2020). Such stressors are likely to impact human performance and cannot be seen as separate from the influence of non-terrestrial gravity (LaGoy et al., 2020).

## Experiencing non-terrestrial gravities

Spaceflight is the ultimate zero gravity environment. During spaceflight, astronauts undergo extreme changes in gravitational exposure. The International Space Station (ISS) is a unique platform for scientific research. The varied length of space missions enables short- and long-term effects of non-terrestrial gravity to be investigated. However, only 600 human beings have been to space so far. The cost associated with launching and the extreme environmental challenges limit the number of individuals that experience “true” zero gravity conditions.

Simulating non-terrestrial gravities on Earth is extremely challenging, but not entirely impossible. Space science methods have allowed the investigation of the effects of non-terrestrial gravity on the body and brain in terrestrial settings. These methods include parabolic flight (Shelhamer & Shelhamer, 2016), centrifugation (Clément et al., 2015), and Head-Down Bed Rest (HDBR; Hargens & Vico, 2016; Pandiarajan & Hargens, 2020; Watenpaugh, 2016). Parabolic Flights enable very brief periods of non-terrestrial gravity to be elicited. The steep acceleration of an aircraft is used to create a 1.8*g* gravitational environment (*hypergravity*) inside the aircraft. The acceleration is then reduced to create a free fall in which the gravity in the aircraft is near weightlessness (0*g*, microgravity). In the final phase, another period of acceleration is experienced to generate 1.8*g* hypergravity. This describes one parabola in which around 20s of hypergravity and 22s of microgravity are induced. The number of parabolas can vary from around 20–30 per parabolic flight campaign. Although non-terrestrial gravity can be experienced, the exposure time is extremely short, limiting the experiments that can be realistically conducted. Human centrifugation enables the generation of short periods of hypergravity. The human centrifuge has been largely used for training purposes and the development of weightlessness countermeasures. For instance, the use of intermittent or continuous centrifugation has been proposed to mitigate the physiological (Clément et al., 2015; Martino et al., 2021) and cognitive (Basner, Dinges, et al., 2021a) impairment triggered by microgravity. Finally, HDBR involves passively tilting participants in a horizontal position for prolonged periods of time. The redistribution of fluids to the head during HDBR is similar to that seen in spaceflight (Mulavara et al., 2018; Ong et al., 2021), and therefore HDBR has been accepted as an effective space analogue. Changes to musculoskeletal, sensorimotor, neurovestibular, and cardiovascular functioning as well as circadian rhythms during HDBR have been noted to mirror spaceflight (Konda et al., 2019; Solbiati et al., 2021; Watenpaugh, 2016). While HDBR allows for a good control over variables, this method creates an incongruency rather than a physical absence or alteration in gravity; there are no changes in the gravitational environment but rather fluid shifts inducing changes in head-foot pressure. Other analogous methods, such as MARS500 and MARS150, have also been utilised to mirror isolation and confinement experienced in space. Here, individuals are subjected to prolonged periods of confinement, for example, 520 days, in purpose-built facilities to examine the impact of isolation, communication delays, stress, sleep, and diet (Gemignani et al., 2014; Solcova & Vinokhodova, 2015; Tafforin, 2013; Ushakov et al., 2014). Potential countermeasures for psychological impacts of spaceflight are largely explored using such simulations (Botella et al., 2016; Feichtinger et al., 2012). However, a limited number of crewmembers or highly trained individuals take part in such simulations. and ultimately, they are still subjected to the terrestrial gravitational vector.

So far space research methods have been mainly used to address the physiological changes in cardiovascular, head-foot pressure shifts, sleep cycle, sensorimotor, muscular, and bone degradation experienced during spaceflight rather than creating physical non-terrestrial gravities (Thornton et al., 1987). During spaceflight, astronauts experience extreme atmospheric conditions including altered carbon dioxide concentrations, radiation, inertial load, and so on. These stressors—and their interaction—are difficult to properly simulate on Earth. Recent methods have attempted to simulate some atmospheric conditions, for instance, the elevated carbon dioxide (CO_2_) levels. HDBR was combined with hypoxia by dispensing carbon dioxide in an isolated chamber to mimic the reduced oxygen levels in space. An increasing number of studies are beginning to utilise this innovative approach (Marshall-Goebel et al., 2017) to investigate brain connectivity (McGregor et al., 2021), human performance (Mahadevan et al., 2021), cognitive and sensorimotor changes (Basner, Stahn, et al., 2021b; Lee, Dios, et al., 2019a), alterations in vestibular processing (Hupfeld, Lee, et al., 2020a), sensorimotor adaptation (Banker et al., 2021), visuomotor adaptation (Salazar et al., 2021), and working memory (Salazar et al., 2020). With new advancements in space technology and engineering, suborbital spaceflights may also become a research method used to explore the effects of non-terrestrial gravities on the human body.

## Non-terrestrial gravities impact human brain and behaviour

Several brain regions are involved in building up the 1*g* Internal Model of Gravity via successful integration of vestibular signals and information from other sensory modalities. Projections from the vestibular system travel to the brainstem and cerebellum, and then reach a distributed cortical and subcortical network of brain areas including the somatosensory cortices, right posterior insula, and temporo-parietal junction (Lopez, 2016; Raiser et al., 2020; zu Eulenburg et al., 2012). Visual, proprioceptive, and sensorimotor inputs converge in this widespread vestibular network. The altered vestibular inputs in space may trigger sensory conflicts, which might eventually lead to maladaptive neuroplasticity (Van Ombergen, Laureys, et al., 2017a). Neuroimaging studies have demonstrated a deactivation across somatosensory and visual cortices in astronauts. This has been suggested to be a compensatory adaptation in response to altered vestibular inputs during spaceflight (Hupfeld et al., 2022) or a sensory reweighting (Pechenkova et al., 2019). Similarly, increased white matter in the cerebellum has been reported following spaceflight, suggesting some sort of sensorimotor neuroplasticity which persisted up to seven months post-flight (Jillings et al., 2020). Whether these changes are functional or maladaptive remains unclear (Roy-O’Reilly et al., 2021).

Neuroimaging studies have highlighted structural changes after microgravity exposure, including the narrowing of the central sulcus (Roberts et al., 2017), an upward shift of the brain (Lee, Koppelmans, et al., 2019b; Roberts et al., 2015, 2017), and changes in white matter (Doroshin et al., 2022; Koppelmans, Pasternak, et al., 2017a). Thinning of occipital areas has also been reported in astronauts (Riascos et al., 2019). Increased cerebrospinal fluid, changes in ventricular volume, and changes in intracranial pressure have been described after spaceflight (Hupfeld, McGregor, et al., 2020b; Kramer et al., 2020). Ventricular volume changes (Alperin et al., 2017; Lee et al., 2021; Roberts et al., 2019) possibly due to a reduced reabsorption of cerebrospinal fluid have also been described (Schneider et al., 2013; Smith et al., 2013; Van Ombergen et al., 2019). Ventricular volume changes persisted two years post-flight with astronauts showing enlargement of the ventricles, three times the rate expected from normal ageing (Roberts et al., 2021). The clinical relevance of this change is yet to be determined.

Similar structural brain changes were also demonstrated using HDBR (Lee et al., 2021). Brain regions particularly involved in vestibular processing demonstrated white matter microstructural change (Lee, Koppelmans, 2019 et al., 2019b), while grey matter changes have been identified in the primary somatosensory cortex, motor cortex (Koppelmans et al., 2016), insula, parietal and occipital lobes (Li et al., 2015), and frontal areas (Koppelmans, Bloomberg, et al., 2017b).

In addition to changes in brain structure, brain functional connectivity is affected by exposure to non-terrestrial gravity. Alterations in resting-state functional connectivity were reported between the motor cortex, cerebellum, and default mode network (Demertzi et al., 2016; Van Ombergen, Ombergen, et al., 2017a, 2017b; Zeng, Liao, et al., 2016a). Connectivity changes in the left anterior insular cortex, anterior part of middle cingulate cortex (Zhou et al., 2014), and motor, somatosensory, and vestibular areas (Cassady et al., 2016) along with changes in regional homogeneity (Liao et al., 2013) and low-frequency brain activity (Liao et al., 2012, 2015) were described. Notably, this reorganisation is likely to a direct consequence of reduced vestibular and motor control abilities in microgravity (Zeng, Liao, et al., 2016a, 2016b). Accordingly, changes in motor cortex excitability were observed after prolonged simulation of non-terrestrial gravity (Roberts et al., 2010), indicating reduced neural efficiency (Yuan et al., 2018).

Not surprisingly, these structural and functional brain changes may lead to neurocognitive alterations. Spatial disorientation, perceptual illusions, balance disorders, motion sickness, altered sensorimotor control, and poor cognitive capability have often been reported by astronauts during spaceflight (Clément et al., 2020). Based on the anatomical and functional features of the brain areas affected by non-terrestrial gravity, we hypothesised that altered vestibular-gravitational signals might affect three main domains of neurocognitive function: a *Sensorimotor Domain* which includes pathways for the integration of sensory signals for orientation, perception, and motor control; a *Cognitive Domain* which includes pathways for regulation of attention, executive functions, decision-making, and other higher cognitive functions; finally, a *Socio-Affective Domain* which includes pathways for regulation of social behaviour and emotions.

### Effects of non-terrestrial gravity on the sensorimotor domain

Astronauts experience a range of sensorimotor disturbances, during spaceflight and upon re-entry. About 70% of astronauts suffer from confusion, disorientation, and motion sickness symptoms—the so-called *Space Motion Sickness*—during orbital flight (Davis et al., 1988; Lackner & DiZio, 2005; Lee et al., 2020; Macias et al., 2020; Russomano et al., 2019). Space Motion Sickness symptoms include dizziness, vertigo, headaches, cold sweating, fatigue, nausea, and vomiting. Consequences range from discomfort to severe sensorimotor and cognitive incapacitation, which cause potential problems during re-entry and emergency exits from a spacecraft. The most destabilising effects of Space Motion Sickness last from the first to the fifth day of weightlessness and reoccur within the first 10 days after landing (Reschke et al., 2018; Thornton & Bonato, 2013).

In microgravity, the mismatch between visual, vestibular, and proprioceptive signals leads to deficits in balance, motor abilities, motor coordination, posture, and head-eye movements. The severity and magnitude of these sensorimotor impairments vary greatly across individuals (Seidler et al., 2015). While some functional abilities such as balance and locomotion remain impaired for up to 16 days post-flight (Mulavara et al., 2010; Reschke & Clément, 2018), other dynamic movements (i.e., jumping) remain altered for much longer (Petersen et al., 2017). Compensatory eye reflexes, such as the ocular counter-rolling reflex, triggered by head movements are reduced following spaceflight (Hallgren et al., 2016) possibly due to decreased vestibular otolith functioning (Hallgren et al., 2015). Alterations in gaze control, spontaneous eye movement, and otolith suppression were found nine days after spaceflight (Kornilova et al., 2012). Visual impairment continues unresolved years after returning from space (Mader et al., 2011, 2017; Nelson et al., 2014)—the so-called Spaceflight Associated Neuro-ocular Syndrome (SANS; Lee et al., 2018, 2020).

Hand, arm, and leg coordination is altered in zero gravity. In non-terrestrial gravities, movement accuracy for arm reaching tasks was found to be altered; an overshooting error emerged in hypergravity (1.8*g*), while undershooting appeared in microgravity (0*g*) (Bringoux et al., 2012). Slower movements were also reported when transitioning from hypergravity to microgravity along with altered grip force (Crevecoeur et al., 2014), and significantly less finely tuned movements have been seen in 0*g* (Crevecoeur et al., 2010).

Sensory processing and perception are also impaired in non-terrestrial gravity. For instance, a bias towards the body axis roll tilt in subjective visual vertical tasks has been described in microgravity (Moore et al., 2010) and after spaceflight (Clément & Wood, 2014). Interestingly, the bias in verticality perception was related to the gravity load experienced by participants, with a greater overestimation at higher gravity levels (Clark et al., 2015). A reliance on visual cues increased effective perceptual upright judgements (Jenkin et al., 2011), indicating the reweighting of visual information in perception. When visual cues were absent, astronauts show larger variance in the subjective visual vertical (Harris et al., 2017). In addition to visual cues, at least 0.15*g* is needed to provide orientation information and perceive the perceptual upright (Harris et al., 2014). Deficits in visual distance perception have also been shown in zero gravity. ISS astronauts underestimated distance, perceived objects as taller and depth as shallower (Clément et al., 2013). Perception of physical distance (Clément et al., 2016) and depth-reversible figures was also impaired during parabolic flight (Clement & Demel, 2012), reinforcing the importance of vestibular-gravitational sensory cues for reliable perceptual experiences.

Humans are well-adapted to Earth’s gravity. Therefore, overcoming the strong terrestrial gravitational prior appears to be difficult despite sensory channels signalling non-terrestrial gravitational cues (Jörges & López-Moliner, 2017). Recently, it has been suggested that this gravity prior seems to have a perceptual nature, rather than being the results of semantic knowledge based on our lifelong experience with terrestrial gravity (Gallagher et al., 2020).

### Effects of non-terrestrial gravity on the cognitive domain

The alteration in brain structure and connectivity, especially in the frontal areas, is likely to impact cognitive functioning. Accordingly, alterations in high-level cognition have been observed during and after spaceflight. This was recently demonstrated by the trailblazing NASA’s Twins Study (Garrett-Bakelman et al., 2019) in which the cognitive performance of astronaut Scott Kelly, while he spent 340 days aboard the ISS, was compared with the performance of his identical twin brother, astronaut Mark Kelly, who remained on Earth. Although Scott’s cognitive efficacy was generally good, he exhibited poor learning and decision-making, and slower performance in executive functions and emotion recognition (Garrett-Bakelman et al., 2019). Previous studies have highlighted learning deficits (Messerotti Benvenuti et al., 2011), sub-optimal decision-making, and impaired strategic decision-making (de La Torre, 2014; de La Torre et al., 2012; Grabherr & Mast, 2010; Strangman et al., 2014). It has been suggested that the overall reduced cognitive efficiency may be due to a competition for resources in demanding tasks (Steinberg et al., 2015). The need for greater neurocognitive control was highlighted in a dual finger-tapping task (Yuan et al., 2016). In this task, participants were asked to respond when a target letter appeared while counting the number of times a target colour was shown (Yuan et al., 2016). An increased reaction time between pre and post 70-day HDBR was reported, suggesting reduced neural efficiency. The competition for resources between brain motor and cognitive areas could have led to this effect. Furthermore, prolonged HDBR induces fluid shifts and pressure changes in the body; this is likely to increase demand on motor cortices as well as recalibration of efferent muscles. The ability to perform efficiently dual verbal memory and prospective time task is also compromised in simulated non-terrestrial gravity (Chen et al., 2013). Cognitively demanding tasks require more neurocognitive resources, which may explain the decline in these particular tasks when exposed to non-terrestrial gravities. Importantly, in actual spaceflight, additional factors such as stress provoked by sleep deprivation and increased workload (Jones et al., 2022), may also impact performance in high-load cognitive tasks.

However, non-terrestrial gravity selectively affects some cognitive functions and spares others. It seems plausible that alterations in gravity influence cognitive functions whose neural substrates are reached by vestibular projections. Accordingly, language and working memory seem to be unaffected by microgravity (Zhao et al., 2011). No differences in performance were shown in a two-back task where participants indicate whether the number was larger than the previous and in visual spatial processing for position (Zhao et al., 2011). However, overall slower responses have been observed (Liu, Zhou, et al., 2015a). Similarly, risky decision-making appears unaffected by microgravity. In the Balloon Analogue Risk Taking (BART), participants showed no effects of non-terrestrial gravity simulation via HDBR procedure, despite a significant decrease in ventromedial prefrontal cortex (VMPC) activity (Rao et al., 2014). Critically, the lack of difference was attributed to the all-male sample and their willingness to take risks. No changes were seen in the severity rating of emergency in microgravity; instead, a recency effect was found (Jiang et al., 2013).

### Effects of non-terrestrial gravity on the socio-affective domain

During space missions, astronauts not only deal with non-terrestrial gravity but also face high levels of confinement and isolation. These factors combined with constant stressors from daylight, noise mission workload, boredom, and communication delays can create a stressful environment, potentially affecting socio-affective processing. Neurocognitive, biological, hormonal, and sleep pattern changes have been reported following spaceflight conditions (Bartone et al., 2018; Landon et al., 2019; Pagel & Choukèr, 2016). Confinement also impacts team dynamics and interaction (Bell et al., 2019), which could have fatal consequences for mission success.

In spaceflight analogue environments such as MARS500 and MARS150, in which individuals are confined to an isolated training facility for weeks, changes in perceived level of stress have been reported (Jacubowski et al., 2015; Nicolas & Gushin, 2015; Rai et al., 2012; Rao et al., 2014; Strollo et al., 2014). Mood changes from anxiety, boredom, and excitement have also been frequently observed (Liu et al., 2016) stress marked by cortisol have been shown to be higher in isolation (Weber et al., 2019). Critically, the aforementioned studies were performed in analogue environments without any physical changes in gravity.

Spaceflight seems to influence emotion recognition and expression. Emotional flanker tasks showed increased reaction time and decreased galvanic skin responses, indicating slower processing to emotional stimuli after bed rest (Liu et al., 2012). Increased negative emotion and decreased positive emotions after just 10 days of simulated non-terrestrial gravity through HDBR (Stavrou et al., 2015; Liu, Zhou, et al., 2015a) were observed. Increased feelings of fatigue and increased negative affective responses were described followed hypoxic bed rest (Stavrou et al., 2018a). Subjects have also shown reduced cooperation (Wang et al., 2017) after HDBR.

It is important to note that there are a limited number of studies exploring socio-affective processing in non-terrestrial gravity environments using quantitative measures, making it difficult to draw conclusions. The detrimental impact of alterations of gravity on stress, mood decline, and increased fatigue is clear. However, the overreliance on self-report measures for anxiety, depression, and motivation in socio-affective research may be susceptible to desirability biases. A more objective quantitative and implicit approach would be desirable.

## Cognition in zero gravity: diffuse framework vs cascade framework?

Our review of the last 10 years of space research showed no comprehensive view of how gravity influences neurocognitive function. Although evidence suggests gravity contributes to sensorimotor, cognitive, and socio-affective domains, findings remain scattered and inconsistent. No consistent picture of the effects of non-terrestrial gravity on human brain and behaviour emerges. In addition to a lack of standardised approach and no replication of studies, there is a lack of a priori hypotheses and neurocognitive models informing theory.

**Figure 1.**
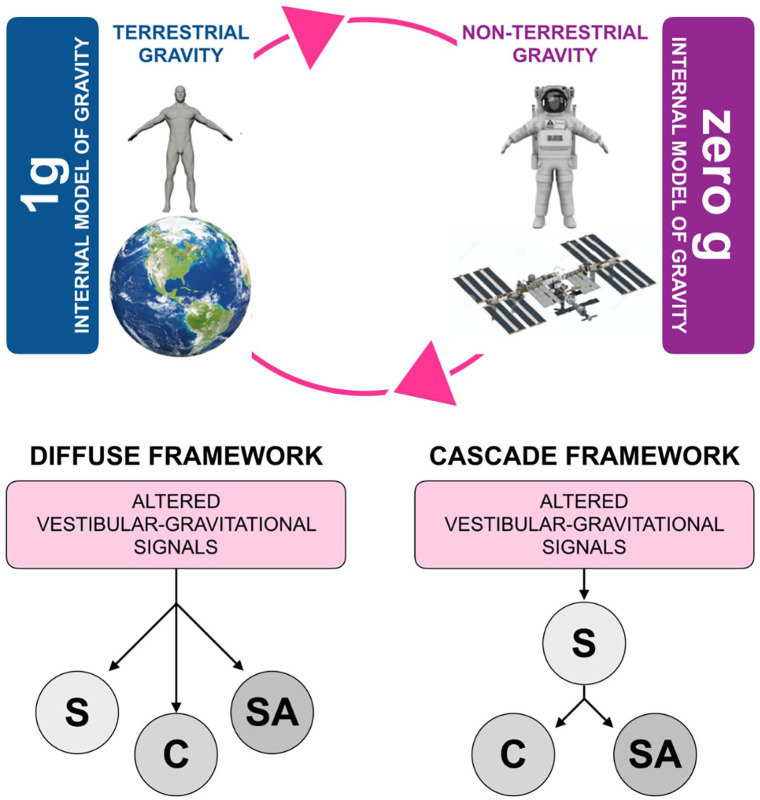
Effects of non-terrestrial gravity on sensorimotor functioning, cognition, and socio-affective processing.

Here, we hypothesised two neurocognitive frameworks to potentially explain the effects of altered gravity on behaviour (Figure 1). First, a *Diffuse Framework* outlines an independent effect of zero gravity on the three domains (Figure 1). That is, deficits in one domain are independent of others. Independent interactions between altered vestibular-gravitational input would occur with each domain. Alternatively, a *Cascade Framework* takes a stepped approach whereby vestibular-gravitational alterations first impact sensorimotor functioning and then cascade onto cognitive and socio-affective processing (Figure 1). According to this model, deficits in sensorimotor functioning, for instance, in arm reaching, may lead to deficits in measures of cognitive processing, such as increased reaction times and slower speeds. This framework suggests a reliance of cognition and socio-affective processing on sensorimotor functioning, and potential bidirectional influences between cognition and socio-affective processing. Thus, a competition for resources may take place whereby the brain is dealing with the unusual sensory information about gravity while performing cognitive and affective tasks. Specifically, the slowed movements in altered gravity environments impact the speed and accuracy of responses to cognitive tasks that often include a key response. A competition for resources may explain this delay, whereby the brain is not only processing unusual sensory information and challenging sensorimotor delays but also competing to process cognitive and affective information.

Critically, both Diffuse and Cascade frameworks highlight a central contribution of vestibular-gravitational signals. On Earth, when the head moves with respect to gravity, the vestibular otoliths shift with the direction of gravitational acceleration, moving the hair cell receptors and signalling to the brain the current gravitational acceleration. Signals from the vestibular organs are integrated with visual and proprioceptive cues, as well as semantic knowledge and past experiences (Lackner & DiZio, 2005; Lacquaniti et al., 2015). Although this internal model is *multimodal*, vestibular cues play a fundamental role. The vestibular otoliths provide information on gravitational acceleration and are directly affected by the lack of gravity in the weightlessness environment. Thus, the vestibular disturbance is key in our conceptualisation.

According with our conceptualisation, Non-terrestrial gravity may affect three key functional domains: sensorimotor functioning (S), cognition (C), and socio-affective processing (SA) (Figure 1). The Diffuse Framework suggests these three domains are implicated independently. The Cascade Framework suggests that sensorimotor functioning is mainly impacted by altered vestibular-gravitational signals and in turn affects cognitive and socio-affective skills.

*But, would it be possible to differentiate between the Diffuse Framework and the Cascade Framework?* We have attempted to tackle this challenge by estimating the *effect size* on a selection of studies in the sensorimotor, cognitive, and socio-affective domains to capture a representative, though clearly *not* systematic sample. Effect sizes are quantitative measures of the magnitude of an experimental effect. They are tied to the magnitude of what has been measured in a study and is used to estimate a specific population parameter, avoiding the arbitrary logic of inferential statistics (i.e., significance testing). Critically, effect sizes are standardised, which makes them independent of a study’s scales and instruments, making it possible to compare different domains and approaches. Here, we reviewed the current literature, and where possible (i.e., when data were available), we estimated the effect sizes of non-terrestrial gravity-induced alterations in sensorimotor, cognitive, and socio-affective functions. We focussed on papers that have been peer-reviewed in the last 10 years, that are widely cited, and that used established methods to simulate non-terrestrial gravity environments. Focus was given to quantitative reports. Studies exploring social factors, culture, group conflict, or team dynamics were not included. Our preliminary search identified approximately 146 articles relevant for sensorimotor domain, 91 articles for the cognitive domain, and 63 for the socio-affective domain. However, for most of these studies, it was not possible to compute the effect size estimates based on the reported statistical details. We have therefore further selected a representative set of studies which are reported in Table 1. We evaluated whether the effect size represents a weak, medium, or strong effect.

**Table 1.** A representative sample of spaceflight studies reporting effects of altered gravity on the sensorimotor, cognitive, and socio-affective domains.

Reference	Function	Non-terrestrial gravity methods	Sample size	Estimated effect size
Sensorimotor domain				
de Witt et al. (2010)	Locomotion	Parabolic flight	5	*d* > 1, *Strong*
Bringoux et al. (2012)	Goal-directed movements	Parabolic flight	8	$\eta_{p}^{2}=.43$ , *Strong*
H. S. Cohen et al. (2012)	Posture	Spaceflight	15	*d* = 1.6, *Strong*
Kornilova et al. (2012)	Gaze control	Spaceflight	26	*d* = 2.81, *Strong*
Lowrey et al. (2014)	Somatosensation	Spaceflight	11	*d* = 1.03, *Strong*
Senot et al. (2012)	Interceptive actions	Parabolic flight	37	*d* > 1, *Strong*
Crevecoeur et al. (2014)	Kinematic	Parabolic flight	12	*d* = 4.2, *Strong*
Ritzmann et al. (2016)	Locomotion	Parabolic flight	8	*d* = 4.08, *Strong*
Cognitive domain				
Messerotti Benvenuti et al. (2011)	Brain plasticity	Head-down bed rest	22	*d* = 0.266, *Weak*
Zhao et al. (2011)	Working memory	Head-down bed rest	44	No effects of gravity, *Weak*
Dalecki et al. (2013)	Mental rotation?	Parabolic flight	6	No effects of gravity, *Weak*
Dolenc & Petrič (2013)	Verbal memory	Head-down bed rest	15	*d* = 0.62, *Medium*
Jiang et al. (2013)	Cognitive judgements	Head-down bed rest	16	No effects of gravity, *Weak*
Basner et al. (2015)	Abstract reasoning	Sleep deprivation	44	*d* = 0.65, *Medium*
Liu, Zhou, et al. (2015a)	Working memory	Head-down bed rest	16	η^2^ = .146, *Medium*
Steinberg et al. (2015)	Control efficacy	Parabolic flight	12	η^2^ = .64, *Strong*
Socio-affective domain				
Rai et al. (2012)	Fatigue	Mars analogue	30	η^2^ = .17, *Strong*
Dern et al. (2014)	Mood and emotion	Altered gravity	16	*d* = .95, *Strong*
Stavrou et al. (2015)	Mood and emotion	Head-down bed rest	11	$\eta_{p}^{2}=.583$ , *Strong*
I. Cohen et al. (2016)	Work performance and cognitive and affective variables	Mars analogue	6	*Weak* (*qualitatively estimated*)
Stavrou et al. (2018a)	Mood and emotion	Head-down bed rest	11	$\eta_{p}^{2}=.28$ , *Strong*
Stavrou et al. (2018b)	Fatigue	Head-down bed rest	11	$\eta_{p}^{2}=.166$ , *Medium*
Weber et al. (2019)	Mood and cognition	Isolation	16	No effects of gravity, *Weak*

Our exercise seems to support the Cascade Framework; the effect sizes are much higher in the sensorimotor domain compared with the cognitive and socio-affective domains highlighting an interesting neurofunctional architecture for the contribution of gravity on behaviour. Importantly, this is aligned with evidence suggesting that there is a strong interaction between vestibular and sensorimotor cues for controlling orientation, posture, and motor control (Lackner & DiZio, 2005).

Here, we have considered studies using different manipulations/simulations of gravity. Studies in the sensorimotor domain adopt spaceflight and parabolic flight, while studies in the cognitive and socio-affective domains mainly use spaceflight, HDBR, and analogue environments. Although our effect size approach is independent of a study’s instruments and sample size, we cannot exclude the possibility that larger effect sizes in sensorimotor studies might reflect a stronger manipulation of gravity. Further research is warranted to quantify the extent to which each domain is affected by different manipulations of non-terrestrial gravity.

Importantly, our exercise has highlighted that the existing space literature is unsystematic in several ways. First, there is a disappointing lack of reporting statistical parameters and effect size in this research field. In some cases, we could identify a single measure which allowed the effect size to be estimated, but in other cases we could not derive a clear estimate of effect size, and therefore many studies were not considered. Second, direct replications are rare in this literature and there is little attempt to validate measures. Although research from the sensorimotor domain recruits a somewhat consistent use of measures, cognitive and socio-affective processing research remains extremely varied. Disappointingly, there is a lack of systematic research exploring changes in socio-affective processing or changes to emotional recognition. The current research relies heavily on self-reported measures that are heavily influenced by desirability biases. Third, across most studies, a lack of control is evident, limiting the conclusion that can be drawn. Without a valid, matched control group, it is difficult to determine whether the cognitive, sensorimotor, or socio-affective changes are indeed due to exposure to an actual or simulated non-terrestrial gravity environment or other confounding variables such as practice effects or stress. Finally, a vast majority of studies lack diversity across their participants. Currently, samples are dominated by young male participants, which is highly problematic because sex and gender differences in neurosensory systems have been largely reported (Reschke et al., 2014). Previous studies have outlined sex and gender differences in adaptation to space (Reschke et al., 2014). Differences in brain activation have also been observed in participants exposed to artificial gravity induced by the centrifuge (Schneider et al., 2014); hypergravity induced increased alpha activity in the frontal cortex for males but not females (Schneider et al., 2014). Similarly, the effectiveness of countermeasures differs between males and females (Macaulay et al., 2016). More generally, differences have also been reported in the anatomy of the peripheral vestibular organs, and women’s susceptibility to vestibular disorders suggests disparities between males and females (Smith et al., 2019) and across a range of physiological aspects including cardiovascular, sensorimotor, and behavioural functions which could impact spaceflight adaptation (Mark et al., 2014).

These considerations lead us to propose a more systematic approach to studying the effects of spaceflight on human behaviour and cognition. A first step involves adopting an interdisciplinary approach (de La Torre et al., 2012; Koppelmans et al., 2013) to gain a more comprehensive view, for instance, combining pre/post-flight neuroimaging methods with in-flight recording of psychophysics (e.g., detection, discrimination, or matching) and quantitative behavioural tasks. Second, testing the same participant across different well-designed and validated tasks might help in understanding the functional effects of non-terrestrial gravity on brain and behaviour. Finally, an open science approach is needed with the provision of experimental data and pre-registration of hypotheses to increase transparency and clarity, pushing the field towards better science practice.

## Conclusion

Having a coherent and accurate perception of the external environment is critical especially during space missions. We have reviewed the effects of non-terrestrial gravity on the human brain and behaviour across the *sensorimotor, cognitive, and socio-affective domains* and have proposed a neurocognitive model based on the effect size of gravity effects on these key functions. The effect sizes are much higher in the sensorimotor domain compared with the cognitive and socio-affective domain, supporting a *Cascade Framework*. Fundamentally, our exercise highlighted the limitations of current human space research. Future studies should take a more systematic approach with a priori hypotheses driven by neurocognitive and neuroanatomical evidence and models. While the methodological challenges of creating physical zero gravity on Earth are inherently insurmountable, generating theoretically driven approaches, recruiting diverse large samples, using a range of tasks across domains, and testing across multiple timepoints can help develop a coherent understanding of the effect of non-terrestrial gravity on the human body and brain. This quantified and systematic approach will not only allow us to identify how gravity constitutes foundational and fundamental signals for cognition, but also enable the development of effective training and interventions for future exciting space exploration, ultimately mitigating against risk.

## References

[bibr1-17470218221113935] Alperin N. Bagci A. M. Lee S. H. (2017). Spaceflight-induced changes in white matter hyperintensity burden in astronauts. Neurology, 89(21), 2187–2191. 10.1212/WNL.0000000000004475 29079684

[bibr2-17470218221113935] Aubert A. E. Larina I. Momken I. Blanc S. White O. Prisk G. K. Linnarsson D. (2016). Towards human exploration of space: The THESEUS review series on cardiovascular, respiratory, and renal research priorities. npj Microgravity, 2(1), 1–9. 10.1038/npjmgrav.2016.31 28725739 PMC5515532

[bibr3-17470218221113935] Banker L. A. Salazar A. P. Lee J. K. Beltran N. E. Kofman I. S. Dios Y. E. D. Mulder E. Bloomberg J. J. Mulavara A. P. Seidler R. D. (2021). The effects of a spaceflight analog with elevated CO2 on sensorimotor adaptation. Journal of Neurophysiology, 125(2), 426–436. 33296611 10.1152/jn.00306.2020

[bibr4-17470218221113935] Barra J. Marquer A. Joassin R. Reymond C. Metge L. Chauvineau V. Pérennou D. (2010). Humans use internal models to construct and update a sense of verticality. Brain, 133(12), 3552–3563. 10.1093/brain/awq311 21097492

[bibr5-17470218221113935] Bartone P. T. Krueger G. P. Bartone J. V. (2018). Individual differences in adaptability to isolated, confined, and extreme environments. Aerospace Medicine and Human Performance, 89(6), 536–546. 10.3357/AMHP.4951.2018 29789087

[bibr6-17470218221113935] Basner M. Dinges D. F. Howard K. Moore T. M. Gur R. C. Mühl C. Stahn A. C. (2021a). Continuous and intermittent artificial gravity as a countermeasure to the cognitive effects of 60 days of head-down tilt bed rest. Frontiers in Physiology, 12, Article 280. 10.3389/fphys.2021.643854 PMC8009974 33815148

[bibr7-17470218221113935] Basner M. Savitt A. Moore T. M. Port A. M. Mcguire S. Ecker A. J. Nasrini J. Mollicone D. J. Mott C. M. Mccann T. Dinges D. F. Gur R. C. (2015). Development and validation of the Cognition test battery for spaceflight. Aerospace Medicine and Human Performance, 86(11), 942–952. 10.3357/AMHP.4343.2015 26564759 PMC4691281

[bibr8-17470218221113935] Basner M. Stahn A. C. Nasrini J. Dinges D. F. Moore T. M. Gur R. C. Muhl C. Macias B. R. Laurie S. S. (2021b). Effects of head-down tilt bed rest plus elevated CO2 on cognitive performance. Journal of Applied Physiology, 130(4), 1235–1246. 10.1152/JAPPLPHYSIOL.00865.2020 33630672 PMC8262780

[bibr9-17470218221113935] Bell S. T. Brown S. G. Mitchell T. (2019). What we know about team dynamics for long-distance space missions: A systematic review of analog research. Frontiers in Psychology, 10, Article 811. 10.3389/fpsyg.2019.00811 PMC6530432 31156490

[bibr10-17470218221113935] Botella C. Baños R. M. Etchemendy E. García-Palacios A. Alcañiz M. (2016). Psychological countermeasures in manned space missions: “EARTH” system for the Mars-500 project. Computers in Human Behavior, 55, 898–908. 10.1016/J.CHB.2015.10.010

[bibr11-17470218221113935] Boyle R. Varelas J. (2021). Otoconia structure after short- and long-duration exposure to altered gravity. Journal of the Association for Research in Otolaryngology, 22(5), 509–525. 10.1007/S10162-021-00791-6 34008038 PMC8476704

[bibr12-17470218221113935] Brenner E. Rodriguez I. A. Muñoz V. E. Schootemeijer S. Mahieu Y. Veerkamp K. Zandbergen M. van der Zee T. Smeets J. B. J. (2016). How can people be so good at intercepting accelerating objects if they are so poor at visually judging acceleration? i-Perception, 7(1), 2041669515624317. 10.1177/2041669515624317 27482367 PMC4954742

[bibr13-17470218221113935] Bringoux L. Blouin J. Coyle T. Ruget H. Mouchnino L. (2012). Effect of gravity-like torque on goal-directed arm movements in microgravity. Journal of Neurophysiology, 107(9), 2541–2548. 10.1152/jn.00364.2011 22298835

[bibr14-17470218221113935] Cassady K. Koppelmans V. Reuter-Lorenz P. de Dios Y. Gadd N. Wood S. Castenada R. R. Kofman I. Bloomberg J. Mulavara A. Seidler R. (2016). Effects of a spaceflight analog environment on brain connectivity and behavior. NeuroImage, 141, 18–30. 10.1016/j.neuroimage.2016.07.029 27423254

[bibr15-17470218221113935] Chen S. S. Zhou R. Xiu L. Chen S. S. Chen X. Tan C. (2013). Effects of 45-day -6° head-down bed rest on the time-based prospective memory. Acta Astronautica, 84, 81–87. 10.1016/j.actaastro.2012.10.040

[bibr16-17470218221113935] Clark T. K. Newman M. C. Oman C. M. Merfeld D. M. Young L. R. (2015). Human perceptual overestimation of whole body roll tilt in hypergravity. Journal of Neurophysiology, 113(7), 2062–2077. 10.1152/jn.00095.2014 25540216 PMC4416546

[bibr17-17470218221113935] Clément G. R. Boyle R. D. George K. A. Nelson G. A. Reschke M. F. Williams T. J. Paloski W. H. (2020). Challenges to the central nervous system during human spaceflight missions to Mars. Journal of Neurophysiology, 123(5), 2037–2063. 32292116 10.1152/jn.00476.2019

[bibr18-17470218221113935] Clément G. R. Bukley A. P. Paloski W. H. (2015). Artificial gravity as a countermeasure for mitigating physiological deconditioning during long-duration space missions. Frontiers in Systems Neuroscience, 9, Article 92. 10.3389/fnsys.2015.00092 PMC4470275 26136665

[bibr19-17470218221113935] Clément G. R. Demel M. (2012). Perceptual reversal of bi-stable figures in microgravity and hypergravity during parabolic flight. Neuroscience Letters, 507(2), 143–146. 10.1016/j.neulet.2011.12.006 22188656

[bibr20-17470218221113935] Clément G. R. Loureiro N. Sousa D. Zandvliet A. (2016). Perception of egocentric distance during gravitational changes in parabolic flight. PLOS ONE, 11(7), Article e0159422. 10.1371/journal.pone.0159422 PMC4963113 27463106

[bibr21-17470218221113935] Clément G. R. Skinner A. Lathan C. (2013). Distance and size perception in astronauts during long-duration spaceflight. Life, 3(4), 524–537. 10.3390/life3040524 25369884 PMC4187133

[bibr22-17470218221113935] Clément G. R. Wood S. J. (2014). Rocking or rolling—perception of ambiguous motion after returning from space. PLOS ONE, 9(10), Article e111107. 10.1371/journal.pone.0111107 PMC4213005 25354042

[bibr23-17470218221113935] Cohen H. S. Kimball K. T. Mulavara A. P. Bloomberg J. J. Paloski W. H. (2012). Posturography and locomotor tests of dynamic balance after long-duration spaceflight. Journal of Vestibular Research: Equilibrium and Orientation, 22(4), 191–196. 10.3233/VES-2012-0456 23142833 PMC8080311

[bibr24-17470218221113935] Cohen I. den Braber N. Smets N. J. van Diggelen J. Brinkman W. P. Neerincx M. A. (2016). Work content influences on cognitive task load, emotional state and performance during a simulated 520-days’ Mars mission. Computers in Human Behavior, 55, 642–652.

[bibr25-17470218221113935] Crevecoeur F. McIntyre J. Thonnard J. L. Lefèvre P. (2010). Movement stability under uncertain internal models of dynamics. Journal of Neurophysiology, 104(3), 1301–1313. 10.1152/jn.00315.2010 20554851

[bibr26-17470218221113935] Crevecoeur F. McIntyre J. Thonnard J.-L. Lefèvre P. (2014). Gravity-dependent estimates of object mass underlie the generation of motor commands for horizontal limb movements. Journal of Neurophysiology, 112(2), 384–392. 10.1152/jn.00061.2014 24790173

[bibr27-17470218221113935] Cullen K. E. (2019). Vestibular processing during natural self-motion: Implications for perception and action. Nature Reviews Neuroscience, 20(6), 346–363. 10.1038/S41583-019-0153-1 30914780 PMC6611162

[bibr28-17470218221113935] Dalecki M. Dern S. Steinberg F. (2013). Mental rotation of a letter, hand and complex scene in microgravity. Neuroscience Letters, 533(1), 55–59. 10.1016/j.neulet.2012.11.002 23147120

[bibr29-17470218221113935] Davis J. R. Vanderploeg J. M. Santy P. A. Jennings R. T. Stewart D. F. (1988). Space motion sickness during 24 flights of the space shuttle. Aviation Space and Environmental Medicine, 59(12), 1185–1189. 3240221

[bibr30-17470218221113935] de La Torre G. G. (2014). Cognitive neuroscience in space. Life, 4(3), 281–294. 10.3390/life4030281 25370373 PMC4206847

[bibr31-17470218221113935] de La Torre G. G. van Baarsen B. Ferlazzo F. Kanas N. Weiss K. Schneider S. Whiteley I. (2012). Future perspectives on space psychology: Recommendations on psychosocial and neurobehavioural aspects of human spaceflight. Acta Astronautica, 81(2), 587–599. 10.1016/j.actaastro.2012.08.013

[bibr32-17470218221113935] Demertzi A. van Ombergen A. Tomilovskaya E. Jeurissen B. Pechenkova E. di Perri C. Litvinova L. Amico E. Rumshiskaya A. Rukavishnikov I. Sijbers J. Sinitsyn V. Kozlovskaya I. B. Sunaert S. Parizel P. M. van de Heyning P. H. Laureys S. Wuyts F. L. (2016). Cortical reorganization in an astronaut’s brain after long-duration spaceflight. Brain Structure and Function, 221(5), 2873–2876. 10.1007/s00429-015-1054-3 25963710 PMC4884200

[bibr33-17470218221113935] Dern S. Vogt T. Abeln V. Strüder H. K. Schneider S. (2014). Psychophysiological responses of artificial gravity exposure to humans. European Journal of Applied Physiology, 114(10), 2061–2071. 10.1007/s00421-014-2927-5 24934228

[bibr34-17470218221113935] de Sá Teixeira N. A. Hecht H. Oliveira A. M . (2013). The representational dynamics of remembered projectile locations. Journal of Experimental Psychology: Human Perception and Performance, 39(6), 1690–1699. 10.1037/a0031777 23398260

[bibr35-17470218221113935] de Witt J. K. Perusek G. P. Lewandowski B. E. Gilkey K. M. Savina M. C. Samorezov S. Edwards W. B . (2010). Locomotion in simulated and real microgravity: Horizontal suspension vs. parabolic flight. Aviation Space and Environmental Medicine, 81(12), 1092–1099. 10.3357/ASEM.2413.2010 21197853

[bibr36-17470218221113935] Dolenc P. Petrič M. (2013). The effects of prolonged physical inactivity induced by bed rest on cognitive functioning in healthy male participants. Annales Kinesiologiae, 4(2), 129–143. http://194.249.2.56/index.php/AK/article/view/13

[bibr37-17470218221113935] Doroshin A. Jillings S. Jeurissen B. Tomilovskaya E. Pechenkova E. Nosikova I. Rumshiskaya A. Litvinova L. Rukavishnikov I. Laet C. D. Schoenmaekers C. Sijbers J. Laureys S. Petrovichev V. Ombergen A. V. Annen J. Sunaert S. Parizel P. M. Sinitsyn V. . . . Wuyts F. L. (2022). Brain connectometry changes in space travelers after long-duration spaceflight. Frontiers in Neural Circuits, 16, Article 815838. 10.3389/FNCIR.2022.815838 PMC8894205 35250494

[bibr38-17470218221113935] Feichtinger E. Charles R. Urbina D. Sundblad P. Fuglesang C. Zell M. (2012, March 3–10). Mars-500—A testbed for psychological crew support during future human exploration missions [Conference session]. IEEE Aerospace Conference Proceedings, Big Sky, MT, United States. 10.1109/AERO.2012.6187396

[bibr39-17470218221113935] Gallagher M. Kearney B. Ferrè E. R. (2021). Where is my hand in space? The internal model of gravity influences proprioception. Biology Letters, 17(6), 20210115. 10.1098/RSBL.2021.0115 34062087 PMC8169209

[bibr40-17470218221113935] Gallagher M. Torok A. Klaas J. Ferrè E. R. (2020). Gravity prior in human behaviour: A perceptual or semantic phenomenon? Experimental Brain Research, 238(9), 1957–1962. 10.1007/s00221-020-05852-5 32567030 PMC7438378

[bibr41-17470218221113935] Garrett-Bakelman F. E. Darshi M. Green S. J. Gur R. C. Lin L. Macias B. R. McKenna M. J. Meydan C. Mishra T. Nasrini J. Piening B. D. Rizzardi L. F. Sharma K. Siamwala J. H. Taylor L. Vitaterna M. H. Afkarian M. Afshinnekoo E. Ahadi S. . . . Turek F. W. (2019). The NASA twins study: A multidimensional analysis of a year-long human spaceflight. Science, 364(6436), Article eaau8650. 10.1126/science.aau8650 PMC7580864 30975860

[bibr42-17470218221113935] Gemignani A. Piarulli A. Menicucci D. Laurino M. Rota G. Mastorci F. Gushin V. Shevchenko O. Garbella E. Pingitore A. Sebastiani L. Bergamasco M. L’Abbate A. Allegrini P. Bedini R. (2014). How stressful are 105days of isolation? Sleep EEG patterns and tonic cortisol in healthy volunteers simulating manned flight to Mars. International Journal of Psychophysiology, 93(2), 211–219. 10.1016/j.ijpsycho.2014.04.008 24793641

[bibr43-17470218221113935] Grabherr L. Mast F. W. (2010). Effects of microgravity on cognition: The case of mental imagery. Article in Journal of Vestibular Research, 20, 53–60. 10.3233/VES-2010-0364 20555167

[bibr44-17470218221113935] Green A. M. Shaikh A. G. Angelaki D. E. (2005). Sensory vestibular contributions to constructing internal models of self-motion. Journal of Neural Engineering, 2(3), S164. 10.1088/1741-2560/2/3/S02 16135882

[bibr45-17470218221113935] Hallgren E. Migeotte P. F. Kornilova L. Delière Q. Fransen E. Glukhikh D. Moore S. T. Clément G. Diedrich A. MacDougall H. Wuyts F. L. (2015). Dysfunctional vestibular system causes a blood pressure drop in astronauts returning from space. Scientific Reports, 5(1), 17627. 26671177 10.1038/srep17627 PMC4680856

[bibr46-17470218221113935] Hallgren E. Kornilova L. Fransen E. Glukhikh D. Moore S. T. Clément G. van Ombergen A. MacDougall H. Naumov I. Wuyts F. L. (2016). Decreased otolith-mediated vestibular response in 25 astronauts induced by long-duration spaceflight. Journal of Neurophysiology, 115(6), 3045–3051. 10.1152/jn.00065.2016 27009158 PMC4922620

[bibr47-17470218221113935] Hargens A. R. Vico L. (2016). Long-duration bed rest as an analog to microgravity. Journal of Applied Physiology, 120(8), 891–903. 26893033 10.1152/japplphysiol.00935.2015

[bibr48-17470218221113935] Harris L. R. Herpers R. Hofhammer T. Jenkin M. (2014). How much gravity is needed to establish the perceptual upright? PLOS ONE, 9(9), Article e106207. 10.1371/journal.pone.0106207 PMC4153541 25184481

[bibr49-17470218221113935] Harris L. R. Jenkin M. Jenkin H. Zacher J. E. Dyde R. T. (2017). The effect of long-term exposure to microgravity on the perception of upright. npj Microgravity, 3(1), 3. 10.1038/s41526-016-0005-5 28649625 PMC5445609

[bibr50-17470218221113935] Hupfeld K. E. Lee J. K. Gadd N. E. Kofman I. S. Dios Y. E. D. Bloomberg J. J. Mulavara A. P. Seidler R. D. (2020a). Neural correlates of vestibular processing during a spaceflight analog with elevated carbon dioxide (CO2): A pilot study. Frontiers in Systems Neuroscience, 13, 80. 10.3389/fnsys.2019.00080 31998084 PMC6965349

[bibr51-17470218221113935] Hupfeld K. E. McGregor H. R. Koppelmans V. Beltran N. E. Kofman I. S. Dios Y. E. D. Riascos R. F. Reuter-Lorenz P. A. Wood S. J. Bloomberg J. J. Mulavara A. P. Seidler R. D. (2022). Brain and behavioral evidence for reweighting of vestibular inputs with long-duration spaceflight. Cerebral Cortex, 32(4), 755–769. 10.1093/CERCOR/BHAB239 34416764 PMC8841601

[bibr52-17470218221113935] Hupfeld K. E. McGregor H. R. Lee J. K. Beltran N. E. Kofman I. S. Dios Y. E. D. Reuter-Lorenz P. A. Riascos R. F. Pasternak O. Wood S. J. Bloomberg J. J. Mulavara A. P. Seidler R. D. Initiative A. D. N. (2020b). The impact of 6 and 12 months in space on human brain structure and intracranial fluid shifts. Cerebral Cortex Communications, 1(1), 1–15. 10.1093/TEXCOM/TGAA023 PMC7446230 32864615

[bibr53-17470218221113935] Hupfeld K. E. McGregor H. R. Reuter-Lorenz P. A. Seidler R. D. (2021). Microgravity effects on the human brain and behavior: Dysfunction and adaptive plasticity. Neuroscience & Biobehavioral Reviews, 122, 176–189. 33454290 10.1016/j.neubiorev.2020.11.017 PMC9650717

[bibr54-17470218221113935] Indovina I. Maffei V. Bosco G. Zago M. Macaluso E. Lacquaniti F. (2005). Representation of visual gravitational motion in the human vestibular cortex. Science, 308(5720), 416–419. 10.1126/science.1107961 15831760

[bibr55-17470218221113935] Jacubowski A. Abeln V. Vogt T. Yi B. Choukèr A. Fomina E. Strüder H. K. Schneider S. (2015). The impact of long-term confinement and exercise on central and peripheral stress markers. Physiology & Behavior, 152, 106–111. 10.1016/j.physbeh.2015.09.017 26387624

[bibr56-17470218221113935] Jenkin M. R. Dyde R. T. Jenkin H. L. Zacher J. E. Harris L. R. (2011). Perceptual upright: The relative effectiveness of dynamic and static images under different gravity states. Seeing and Perceiving, 24(1), 53–64. 10.1163/187847511X555292 21406155

[bibr57-17470218221113935] Jiang C.-M. M. Zheng R. Zhou Y. Liang Z.-Y. Y. Rao L.-L. L. Sun Y. Tan C. Chen X.-P. P. Tian Z.-Q. Q. Bai Y.-Q. Q. Chen S.-G. G. Li S. (2013). Effect of 45-day simulated microgravity on the evaluation of orally reported emergencies. Ergonomics, 56(8), 1225–1231. 10.1080/00140139.2013.809481 23789793

[bibr58-17470218221113935] Jillings S. Ombergen A. V. Tomilovskaya E. Rumshiskaya A. Litvinova L. Nosikova I. Pechenkova E. Rukavishnikov I. Kozlovskaya I. B. Manko O. Danilichev S. Sunaert S. Parizel P. M. Sinitsyn V. Petrovichev V. Laureys S. Eulenburg P. Sijbers J. Wuyts F. L. Jeurissen B. (2020). Macro- and microstructural changes in cosmonauts’ brains after long-duration spaceflight. Science Advances, 6(36), Article eaaz9488. 10.1126/sciadv.aaz9488 PMC7473746 32917625

[bibr59-17470218221113935] Jones C. W. Basner M. Mollicone D. J. Mott C. M. Dinges D. F. (2022). Sleep deficiency in spaceflight is associated with degraded neurobehavioral functions and elevated stress in astronauts on six-month missions aboard the International Space Station. Sleep, 45(3), Article zsac006. 10.1093/SLEEP/ZSAC006 PMC8919197 35023565

[bibr60-17470218221113935] Jörges B. López-Moliner J. (2017). Gravity as a strong prior: Implications for perception and action. Frontiers in Human Neuroscience, 11, Article 203. 10.3389/fnhum.2017.00203 PMC5408029 28503140

[bibr61-17470218221113935] Kanas N. Manzey D. (2008). Space psychology and psychiatry. Springer. 10.1007/978-1-4020-6770-9

[bibr62-17470218221113935] Konda N. N. Karri R. S. Winnard A. Nasser M. Evetts S. Boudreau E. Caplan N. Gradwell D. Velho R. M. (2019). A comparison of exercise interventions from bed rest studies for the prevention of musculoskeletal loss. npj Microgravity, 2019(1), 5151–5111. 10.1038/s41526-019-0073-4 PMC6506471 31098391

[bibr63-17470218221113935] Koppelmans V. Bloomberg J. J. de Dios Y. E. Wood S. J. Reuter-Lorenz P. A. Kofman I. S. Riascos R. Mulavara A. P. Seidler R. D. (2017b). Brain plasticity and sensorimotor deterioration as a function of 70 days head down tilt bed rest. PLOS ONE, 12(8), Article e0182236. 10.1371/journal.pone.0182236 PMC5540603 28767698

[bibr64-17470218221113935] Koppelmans V. Erdeniz B. de Dios Y. E. Wood S. J. Reuter-Lorenz P. A. Kofman I. Bloomberg J. J. Mulavara A. P. Seidler R. D. (2013). Study protocol to examine the effects of spaceflight and a spaceflight analog on neurocognitive performance: Extent, longevity, and neural bases. BMC Neurology, 13(1), Article 205. 10.1186/1471-2377-13-205 PMC3878338 24350728

[bibr65-17470218221113935] Koppelmans V. Pasternak O. Bloomberg J. J. Dios Y. E. D. Wood S. J. Riascos R. Reuter-Lorenz P. A. Kofman I. S. Mulavara A. P. Seidler R. D. (2017a). Intracranial fluid redistribution but no white matter microstructural changes during a spaceflight analog. Scientific Reports, 7(1), 3154. 10.1038/s41598-017-03311-w 28600534 PMC5466616

[bibr66-17470218221113935] Koppelmans V. Seidler R. Bloomberg J. Mulavara A. (2016). Brain structural plasticity with spaceflight. npj Microgravity, 2(1), 2. 10.1038/s41526-016-0001-9 28649622 PMC5460234

[bibr67-17470218221113935] Kornilova L. N. Naumov I. A. Azarov K. A. Sagalovitch V. N. (2012). Gaze control and vestibular-cervical-ocular responses after prolonged exposure to microgravity. Aviation Space and Environmental Medicine, 83(12), 1123–1134. 10.3357/ASEM.3106.2012 23316540

[bibr68-17470218221113935] Kramer L. A. Hasan K. M. Stenger M. B. Sargsyan A. Laurie S. S. Otto C. Ploutz-Snyder R. J. Marshall-Goebel K. Riascos R. F. Macias B. R. (2020). Intracranial effects of microgravity: A prospective longitudinal MRI study. Radiology, 295(3), 640–648. 32286194 10.1148/radiol.2020191413

[bibr69-17470218221113935] Lackner J. R. DiZio P. (2005). Vestibular, proprioceptive, and haptic contributions to spatial orientation. Annual Review of Psychology, 56, 115–147. 10.1146/annurev.psych.55.090902.142023 15709931

[bibr70-17470218221113935] Lacquaniti F. Bosco G. Gravano S. Indovina I. Scaleia B. L. Maffei V. Zago M. (2015). Gravity in the brain as a reference for space and time perception. Multisensory Research, 28(5–6), 397–426. 10.1163/22134808-00002471 26595949

[bibr71-17470218221113935] Lacquaniti F. Maioli C. (1989). Adaptation to suppression of visual information during catching. Journal of Neuroscience, 9(1), 149–159. 10.1523/jneurosci.09-01-00149.1989 2913201 PMC6570000

[bibr72-17470218221113935] LaGoy A. D. Sinnott A. M. Ambarian M. Pepping G. J. Simpson R. J. Agha N. H. Bower J. L. Alfano C. A. Connaboy C. (2020). Differences in affordance-based behaviors within an isolated and confined environment are related to sleep, emotional health and physiological parameters. Acta Astronautica, 176, 238–246. 10.1016/j.actaastro.2020.06.034

[bibr73-17470218221113935] Landon L. B. Douglas G. L. Downs M. E. Greene M. R. Whitmire A. M. Zwart S. R. Roma P. G. (2019). The behavioral biology of teams: Multidisciplinary contributions to social dynamics in isolated, confined, and extreme environments. Frontiers in Psychology, 10, Article 2571. 10.3389/fpsyg.2019.02571 PMC6883946 31824374

[bibr74-17470218221113935] Lang T. van Loon J. J. W. A. Bloomfield S. Vico L. Chopard A. Rittweger J. Kyparos A. Blottner D. Vuori I. Gerzer R. Cavanagh P. R. (2017). Towards human exploration of space: The THESEUS review series on muscle and bone research priorities. npj Microgravity, 3(1), 1–10. 10.1038/s41526-017-0013-0 28649630 PMC5445590

[bibr75-17470218221113935] Lee A. G. Mader T. H. Gibson C. R. Brunstetter T. J. Tarver W. J. (2018). Space flight-associated neuro-ocular syndrome (SANS). Eye, 32(7), 1164–1167. 29527011 10.1038/s41433-018-0070-y PMC6043524

[bibr76-17470218221113935] Lee A. G. Mader T. H. Gibson C. R. Tarver W. Rabiei P. Riascos R. F. Galdamez L. A. Brunstetter T. (2020). Spaceflight associated neuro-ocular syndrome (SANS) and the neuro-ophthalmologic effects of microgravity: A review and an update. npj Microgravity, 6(1), 1–10. 10.1038/s41526-020-0097-9 32047839 PMC7005826

[bibr77-17470218221113935] Lee J. K. Dios Y. D. Kofman I. Mulavara A. P. Bloomberg J. J. Seidler R. D. (2019a). Head down tilt bed rest plus elevated CO2 as a spaceflight analog: Effects on cognitive and sensorimotor performance. Frontiers in Human Neuroscience, 13, Article 355. 10.3389/fnhum.2019.00355 PMC6811492 31680909

[bibr78-17470218221113935] Lee J. K. Koppelmans V. Pasternak O. Beltran N. E. Kofman I. S. Dios Y. E. D. Mulder E. R. Mulavara A. P. Bloomberg J. J. Seidler R. D. Fixel N. (2021). Effects of spaceflight stressors on brain volume, microstructure, and intracranial fluid distribution. Cerebral Cortex Communications, 2(2), 1–14. 10.1093/TEXCOM/TGAB022 PMC8152913 34296167

[bibr79-17470218221113935] Lee J. K. Koppelmans V. Riascos R. F. Hasan K. M. Pasternak O. Mulavara A. P. Bloomberg J. J. Seidler R. D. (2019b). Spaceflight-associated brain white matter microstructural changes and intracranial fluid redistribution. JAMA Neurology, 76(4), 412–419. 10.1001/jamaneurol.2018.4882 30673793 PMC6459132

[bibr80-17470218221113935] Li K. Guo X. Jin Z. Ouyang X. Zeng Y. Feng J. Wang Y. Yao L. Ma L. (2015). Effect of simulated microgravity on human brain gray matter and white matter—Evidence from MRI. PLOS ONE, 10(8), Article e0135835. 10.1371/journal.pone.0135835 PMC4535759 26270525

[bibr81-17470218221113935] Liao Y. Lei M. Huang H. Wang C. Duan J. Li H. Liu X. (2015). The time course of altered brain activity during 7-day simulated microgravity. Frontiers in Behavioral Neuroscience, 9, Article 124. 10.3389/fnbeh.2015.00124 PMC4428138 26029071

[bibr82-17470218221113935] Liao Y. Miao D. Huan Y. Yin H. Xi Y. Liu X. (2013). Altered regional homogeneity with short-term simulated microgravity and its relationship with changed performance in mental transformation. PLOS ONE, 8(6), Article e64931. 10.1371/journal.pone.0064931 PMC3670926 23755162

[bibr83-17470218221113935] Liao Y. Zhang J. Huang Z. Xi Y. Zhang Q. Zhu T. Liu X. (2012). Altered baseline brain activity with 72 h of simulated microgravity—Initial evidence from resting-state fMRI. PLOS ONE, 7(12), Article e52558. 10.1371/journal.pone.0052558 PMC3528642 23285086

[bibr84-17470218221113935] Liu Q. Zhou R. Chen S. Tan C. (2012). Effects of head-down bed rest on the executive functions and emotional response. PLOS ONE, 7(12), Article e52160. 10.1371/journal.pone.0052160 PMC3524097 23284916

[bibr85-17470218221113935] Liu Q. Zhou R. Liu L. Zhao X. (2015b). Effects of 72 hours total sleep deprivation on male astronauts’ executive functions and emotion. Comprehensive Psychiatry, 61, 28–35. 26112064 10.1016/j.comppsych.2015.05.015

[bibr86-17470218221113935] Liu Q. Zhou R. Zhao X. Soei T. P. S. (2015a). Effects of prolonged head-down bed rest on working memory. Neuropsychiatric Disease and Treatment, 11, 835–842. 10.2147/NDT.S76292 25848281 PMC4381883

[bibr87-17470218221113935] Liu Q. Zhou R. L. Zhao X. Chen X. P. Chen S. G. (2016). Acclimation during space flight: Effects on human emotion. Military Medical Research, 3(1), 1–5. 10.1186/S40779-016-0084-3 27134755 PMC4850672

[bibr88-17470218221113935] Lopez C. (2016). The vestibular system: Balancing more than just the body. Current Opinion in Neurology, 29(1), 74–83. 10.1097/WCO.0000000000000286 26679566

[bibr89-17470218221113935] Lowrey C. R. Perry S. D. Strzalkowski N. D. J. Williams D. R. Wood S. J. Bent L. R. (2014). Selective skin sensitivity changes and sensory reweighting following short-duration space flight. Journal of Applied Physiology, 116(6), 683–692. 10.1152/japplphysiol.01200.2013 24458748

[bibr90-17470218221113935] Macaulay T. R. Macias B. R. Lee S. M. C. Boda W. L. Watenpaugh D. E. Hargens A. R. (2016). Treadmill exercise within lower-body negative pressure attenuates simulated spaceflight-induced reductions of balance abilities in men but not women. npj Microgravity, 2(1), 16022. 10.1038/npjmgrav.2016.22 28725733 PMC5515523

[bibr91-17470218221113935] Macias B. R. Patel N. B. Gibson C. R. Samuels B. C. Laurie S. S. Otto C. Ferguson C. R. Lee S. M. C. Ploutz-Snyder R. Kramer L. A. Mader T. H. Brunstetter T. Stenger M. B. (2020). Association of long-duration spaceflight with anterior and posterior ocular structure changes in astronauts and their recovery. JAMA Ophthalmology, 138(5), 553–559. 10.1001/JAMAOPHTHALMOL.2020.0673 32239198 PMC7118682

[bibr92-17470218221113935] Mader T. H. Gibson C. R. Otto C. A. Sargsyan A. E. Miller N. R. Subramanian P. S. Hart S. F. Lipsky W. Patel N. B. Lee A. G. (2017). Persistent asymmetric optic disc swelling after long-duration space flight: Implications for pathogenesis. Journal of Neuro-Ophthalmology, 37(2), 133–139. 10.1097/WNO.0000000000000467 27930421

[bibr93-17470218221113935] Mader T. H. Gibson C. R. Pass A. F. Kramer L. A. Lee A. G. Fogarty J. Tarver W. J. Dervay J. P. Hamilton D. R. Sargsyan A. Phillips J. L. Tran D. Lipsky W. Choi J. Stern C. Kuyumjian R. Polk J. D. (2011). Optic disc edema, globe flattening, choroidal folds, and hyperopic shifts observed in astronauts after long-duration space flight. Ophthalmology, 118(10), 2058–2069. 10.1016/j.ophtha.2011.06.021 21849212

[bibr94-17470218221113935] Mahadevan A. D. Hupfeld K. E. Lee J. K. Dios Y. E. D. Kofman I. S. Beltran N. E. Mulder E. Bloomberg J. J. Mulavara A. P. Seidler R. D. (2021). Head-down-tilt bed rest with elevated CO2: Effects of a pilot spaceflight analog on neural function and performance during a cognitive-motor dual task. Frontiers in Physiology, 12, Article 654906. 10.3389/FPHYS.2021.654906/FULL PMC8424013 34512371

[bibr95-17470218221113935] Mark S. Scott G. B. I. Donoviel D. B. Leveton L. B. Mahoney E. Charles J. B. Siegel B. (2014). The impact of sex and gender on adaptation to space: Executive summary. Journal of Women’s Health, 23(11), 941–947. 10.1089/jwh.2014.4914 PMC4236030 25401937

[bibr96-17470218221113935] Marshall-Goebel K. Mulder E. Donoviel D. Strangman G. Suarez J. I. Rao C. V. Frings-Meuthen P. Limper U. Rittweger J. Bershad E. M. (2017). An international collaboration studying the physiological and anatomical cerebral effects of carbon dioxide during head-down tilt bed rest: The SPACECOT study. Journal of Applied Physiology, 122(6), 1398–1405. 10.1152/japplphysiol.00885.2016 28235859

[bibr97-17470218221113935] Martino E. de Salomoni S. E. Hodges P. W. Hides J. Lindsay K. Debuse D. Winnard A. Elliott J. Hoggarth M. Beard D. Cook J. A. Ekman R. Hinterwaldner L. Scott J. Weber T. Caplan N. (2021). Intermittent short-arm centrifugation is a partially effective countermeasure against upright balance deterioration following 60-day head-down tilt bed rest. Journal of Applied Physiology, 131(2), 689–701. 34197228 10.1152/japplphysiol.00180.2021

[bibr98-17470218221113935] McGregor H. R. Lee J. K. Mulder E. R. Dios Y. E. D. Beltran N. E. Kofman I. S. Bloomberg J. J. Mulavara A. P. Seidler R. D. (2021). Brain connectivity and behavioral changes in a spaceflight analog environment with elevated CO2. NeuroImage, 225, 117450. 10.1016/j.neuroimage.2020.117450 33075558

[bibr99-17470218221113935] Messerotti Benvenuti S. Bianchin M. Angrilli A. Benvenuti S. Bianchin M. Angrilli A . (2011). Effects of simulated microgravity on brain plasticity: A startle reflex habituation study. Physiology & Behavior, 104(3), 503–506. 10.1016/j.physbeh.2011.05.019 21627974

[bibr100-17470218221113935] Monache S. D. Lacquaniti F. Bosco G. (2019). Ocular tracking of occluded ballistic trajectories: Effects of visual context and of target law of motion. Journal of Vision, 19(4), 13. 10.1167/19.4.13 30952164

[bibr101-17470218221113935] Moore S. T. Macdougall H. G. Paloski W. H. (2010). Effects of head-down bed rest and artificial gravity on spatial orientation. Experimental Brain Research, 204, 617–622. 10.1007/s00221-010-2317-0 20535455

[bibr102-17470218221113935] Mulavara A. P. Feiveson A. H. Fiedler J. Cohen H. Peters B. T. Miller C. Brady R. Bloomberg J. J. (2010). Locomotor function after long-duration space flight: Effects and motor learning during recovery. Experimental Brain Research, 202(3), 649–659. 10.1007/s00221-010-2171-0 20135100

[bibr103-17470218221113935] Mulavara A. P. Peters B. T. Miller C. A. Kofman I. S. Reschke M. F. Taylor L. C. Lawrence E. L. Wood S. J. Laurie S. S. Lee S. M. C. Buxton R. E. May-Phillips T. R. Stenger M. B. Ploutz-Snyder L. L. Ryder J. W. Feiveson A. H. Bloomberg J. J. (2018). Physiological and functional alterations after spaceflight and bed rest. Medicine and Science in Sports and Exercise, 50(9), 1961–1980. 10.1249/MSS.0000000000001615 29620686 PMC6133205

[bibr104-17470218221113935] Nelson E. Mulugeta L. Myers J. (2014). Microgravity-induced fluid shift and ophthalmic changes. Life, 4(4), 621–665. 10.3390/life4040621 25387162 PMC4284461

[bibr105-17470218221113935] Nicolas M. Gushin V. (2015). Stress and recovery responses during a 105-day ground-based space simulation. Stress and Health, 31(5), 403–410. 10.1002/smi.2565 24616284

[bibr106-17470218221113935] Ong J. Lee A. G. Moss H. E. (2021). Head-down tilt bed rest studies as a terrestrial analog for spaceflight associated neuro-ocular syndrome. Frontiers in Neurology, 12, Article 648958. 10.3389/FNEUR.2021.648958 PMC8032981 33841315

[bibr107-17470218221113935] Pagel J. I. Choukèr A. (2016). Effects of isolation and confinement on humans-implications for manned space explorations. Journal of Applied Physiology, 120(12), 1449–1457. 10.1152/japplphysiol.00928.2015 26846554

[bibr108-17470218221113935] Pandiarajan M. Hargens A. R. (2020). Ground-based analogs for human spaceflight. Frontiers in Physiology, 11, Article 716. 10.3389/fphys.2020.00716 PMC7324748 32655420

[bibr109-17470218221113935] Patel Z. S. Brunstetter T. J. Tarver W. J. Whitmire A. M. Zwart S. R. Smith S. M. Huff J. L. (2020). Red risks for a journey to the red planet: The highest priority human health risks for a mission to Mars. npj Microgravity, 6(1), 33. 10.1038/s41526-020-00124-6 33298950 PMC7645687

[bibr110-17470218221113935] Pechenkova E. V. Nosikova I. N. Rumshiskaya A. D. Litvinova L. D. Rukavishnikov I. V. Mershina E. A. Sinitsin V. E. Ombergen A. V. Jeurissen B. Jillings S. D. Laureys S. Sijbers J. Grishin A. Chernikova L. A. Naumov I. A. Kornilova L. N. Wuyts F. L. Tomilovskaya E. S. Kozlovskaya I. B. (2019). Alterations of functional brain connectivity after long-duration spaceflight as revealed by fMRI. Frontiers in Physiology, 10, Article 761. 10.3389/fphys.2019.00761 PMC6621543 31333476

[bibr111-17470218221113935] Petersen N. Lambrecht G. Scott J. Hirsch N. Stokes M. Mester J. (2017). Postflight reconditioning for European Astronauts—A case report of recovery after six months in space. Musculoskeletal Science and Practice, 27, S23–S31. 10.1016/j.msksp.2016.12.010 28173929

[bibr112-17470218221113935] Rai B. Foing B. H. Kaur J. (2012). Working hours, sleep, salivary cortisol, fatigue and neuro-behavior during Mars analog mission: Five crews study. Neuroscience Letters, 516(2), 177–181. 10.1016/j.neulet.2012.03.067 22487731

[bibr113-17470218221113935] Raiser T. M. Flanagin V. L. Duering M. Ombergen A. van Ruehl R. M. zu Eulenburg P. (2020). The human corticocortical vestibular network. NeuroImage, 223, 117362. 10.1016/J.NEUROIMAGE.2020.117362 32919059

[bibr114-17470218221113935] Rao L.-L. Zhou Y. Liang Z.-Y. Rao H. Zheng R. Sun Y. Tan C. Xiao Y. Tian Z.-Q. Chen X.-P. Wang C.-H. Bai Y.-Q. Chen S.-G. Li S. (2014). Decreasing ventromedial prefrontal cortex deactivation in risky decision making after simulated microgravity: Effects of âˆ’6Â° head-down tilt bed rest. Frontiers in Behavioral Neuroscience, 8, Article 187. 10.3389/fnbeh.2014.00187 PMC4034329 24904338

[bibr115-17470218221113935] Reschke M. F. Clément G. (2018). Vestibular and sensorimotor dysfunction during space flight. Current Pathobiology Reports, 6(3), 177–183. 10.1007/s40139-018-0173-y

[bibr116-17470218221113935] Reschke M. F. Cohen H. S. Cerisano J. M. Clayton J. A. Cromwell R. Danielson R. W. Hwang E. Y. Tingen C. Allen J. R. Tomko D. L. (2014). Effects of sex and gender on adaptation to space: Neurosensory systems. Journal of Women’s Health, 23(11), 959–962. 10.1089/jwh.2014.4908 PMC4236059 25401941

[bibr117-17470218221113935] Reschke M. F. Wood S. J. Clément G. R. (2018). A case study of severe space motion sickness. Aerospace Medicine and Human Performance, 89(8), 749–753. 10.3357/AMHP.5071.2018 30020061

[bibr118-17470218221113935] Riascos R. F. Kamali A. Hakimelahi R. Mwangi B. Rabiei P. Seidler R. D. Behzad B. B. Keser Z. Kramer L. A. Hasan K. M. (2019). Longitudinal analysis of quantitative brain MRI in astronauts following microgravity exposure. Journal of Neuroimaging, 29(3), 323–330. 10.1111/JON.12609 30784130

[bibr119-17470218221113935] Ritzmann R. Freyler K. Krause A. Gollhofer A. (2016). Bouncing on Mars and the Moon—The role of gravity on neuromuscular control: Correlation of muscle activity and rate of force development. Journal of Applied Physiology, 121(5), 1187–1195. 10.1152/japplphysiol.00692.2016 27660301

[bibr120-17470218221113935] Roberts D. R. Albrecht M. H. Collins H. R. Asemani D. Chatterjee A. R. Spampinato M. V. Zhu X. Chimowitz M. I. Antonucci M. U. (2017). Effects of spaceflight on astronaut brain structure as indicated on MRI. New England Journal of Medicine, 377(18), 1746–1753. 10.1056/NEJMoa1705129 29091569

[bibr121-17470218221113935] Roberts D. R. Inglesby D. C. Brown T. R. Collins H. R. Eckert M. A. Asemani D. (2021). Longitudinal change in ventricular volume is accelerated in astronauts undergoing long-duration spaceflight. Aging Brain, 1, 100017. 10.1016/J.NBAS.2021.100017 36911514 PMC9997154

[bibr122-17470218221113935] Roberts D. R. Nietert P. Eckert M. George M. Bloomberg J. Asemani D. Inglesby D. Brown T. (2019). Prolonged microgravity affects human brain structure and function. American Journal of Neuroradiology, 40(11), 1878–1885. 10.3174/ajnr.A6249 31624117 PMC6975111

[bibr123-17470218221113935] Roberts D. R. Ramsey D. Johnson K. Kola J. Ricci R. Hicks C. Borckardt J. J. Bloomberg J. J. Epstein C. George M. S. (2010). Cerebral cortex plasticity after 90 days of bed rest: Data from TMS and fMRI. Aviation Space and Environmental Medicine, 81(1), 30–40. 10.3357/ASEM.2532.2009 20058735 PMC2861654

[bibr124-17470218221113935] Roberts D. R. Zhu X. Tabesh A. Duffy E. W. Ramsey D. A. Brown T. R. (2015). Structural brain changes following long-term 6° head-down tilt bed rest as an analog for spaceflight. American Journal of Neuroradiology, 36(11), 2048–2054. 10.3174/ajnr.A4406 26185326 PMC7964872

[bibr125-17470218221113935] Roy-O’Reilly M. Mulavara A. Williams T. (2021). A review of alterations to the brain during spaceflight and the potential relevance to crew in long-duration space exploration. npj Microgravity, 7(1), 5. 10.1038/s41526-021-00133-z 33594073 PMC7887220

[bibr126-17470218221113935] Russomano T. Rosa M. D. Santos M. D. (2019). Space motion sickness: A common neurovestibular dysfunction in microgravity. Neurology India, 67(8), S214–S218. 10.4103/0028-3886.259127 31134912

[bibr127-17470218221113935] Salazar A. P. Hupfeld K. E. Lee J. K. Banker L. A. Tays G. D. Beltran N. E. Kofman I. S. Dios Y. E. D. Mulder E. Bloomberg J. J. Mulavara A. P. Seidler R. D. (2021). Visuomotor adaptation brain changes during a spaceflight analog with elevated carbon dioxide (CO2): A pilot study. Frontiers in Neural Circuits, 15, Article 51. 10.3389/fncir.2021.659557 PMC8215599 34163332

[bibr128-17470218221113935] Salazar A. P. Hupfeld K. E. Lee J. K. Beltran N. E. Kofman I. S. Dios Y. E. D. Mulder E. Bloomberg J. J. Mulavara A. P. Seidler R. D. (2020). Neural working memory changes during a spaceflight analog with elevated carbon dioxide: A pilot study. Frontiers in Systems Neuroscience, 14, Article 48. 10.3389/fnsys.2020.00048 PMC7399639 32848641

[bibr129-17470218221113935] Schneider S. Abeln V. Askew C. D. Vogt T. Hoffmann U. Denise P. Strüder H. K. (2013). Changes in cerebral oxygenation during parabolic flight. European Journal of Applied Physiology, 113(6), 1617–1623. 10.1007/s00421-013-2588-9 23334389

[bibr130-17470218221113935] Schneider S. Robinson R. Smith C. Wiesche M. V. D. Goswami N. (2014). Gender specific changes in cortical activation patterns during exposure to artificial gravity. Acta Astronautica, 104(1), 438–443. 10.1016/J.ACTAASTRO.2014.03.003

[bibr131-17470218221113935] Seidler R. D. Mulavara A. P. Bloomberg J. J. Peters B. T. (2015). Individual predictors of sensorimotor adaptability. Frontiers in Systems Neuroscience, 9, Article 100. 10.3389/fnsys.2015.00100 PMC4491631 26217197

[bibr132-17470218221113935] Senot P. Zago M. le Séac’h A. Zaoui M. Berthoz A. Lacquaniti F. McIntyre J. (2012). When up is down in 0g: How gravity sensing affects the timing of interceptive actions. Journal of Neuroscience, 32(6), 1969–1973. 10.1523/JNEUROSCI.3886-11.2012 22323710 PMC6621712

[bibr133-17470218221113935] Shelhamer M. Shelhamer M. (2016). Parabolic flight as a spaceflight analog. Journal of Applied Physiology, 120(12), 1442–1448. 10.1152/JAPPLPHYS-IOL.01046.2015 26796759

[bibr134-17470218221113935] Smith C. Goswami N. Robinson R. von der Wiesche M. Schneider S. (2013). The relationship between brain cortical activity and brain oxygenation in the prefrontal cortex during hypergravity exposure. Journal of Applied Physiology, 114(7), 905–910. 10.1152/japplphysiol.01426.2012 23372141

[bibr135-17470218221113935] Smith P. F. Agrawa Y. Darlington C. L. (2019). Sexual dimorphism in vestibular function and dysfunction. Journal of Neurophysiology, 121(6), 2379–2391. 31042453 10.1152/jn.00074.2019

[bibr136-17470218221113935] Solbiati S. Martin-Yebra A. Vaïda P. Caiani E. G. (2021). Evaluation of cardiac circadian rhythm deconditioning induced by 5-to-60 days of head-down bed rest. Frontiers in Physiology, 11, Article 1780. 10.3389/fphys.2020.612188 PMC7838678 33519517

[bibr137-17470218221113935] Solcova I. Vinokhodova A. G. (2015). Locus of control, stress resistance, and personal growth of participants in the Mars-500 experiment. Human Physiology, 41(7), 761–766. 10.1134/S0362119715070221

[bibr138-17470218221113935] Stavrou N. A. M. Debevec T. Eiken O. Mekjavic I. B. (2018a). Hypoxia exacerbates negative emotional state during inactivity: The effect of 21 days hypoxic bed rest and confinement. Frontiers in Physiology, 9, Article 26. 10.3389/fphys.2018.00026 PMC5809445 29472866

[bibr139-17470218221113935] Stavrou N. A. M. Debevec T. Eiken O. Mekjavic I. B. (2018b). Hypoxia Worsens Affective Responses and Feeling of Fatigue During Prolonged Bed Rest. Frontiers in Psychology, 9, Article 362. 10.3389/fpsyg.2018.00362 PMC5876302 29628903

[bibr140-17470218221113935] Stavrou N. A. M. McDonnell A. C. Eiken O. Mekjavic I. B. (2015). Psychological strain: Examining the effect of hypoxic bedrest and confinement. Physiology & Behavior, 139, 497–504. 10.1016/j.physbeh.2014.12.015 25484354

[bibr141-17470218221113935] Steinberg F. Kalicinski M. Dalecki M. Bock O. (2015). Human performance in a realistic instrument-control task during short-term microgravity. PLOS ONE, 10(6), Article e0128992. 10.1371/journal.pone.0128992 PMC4470626 26083473

[bibr142-17470218221113935] Strangman G. E. Sipes W. Beven G. (2014). Human cognitive performance in spaceflight and analogue environments. Aviation, Space, and Environmental Medicine, 85(10), 1033–1048. 25245904 10.3357/ASEM.3961.2014

[bibr143-17470218221113935] Strollo F. Vassilieva G. Ruscica M. Masini M. Santucci D. Borgia L. Magni P. Celotti F. Nikiporuc I. (2014). Changes in stress hormones and metabolism during a 105-day simulated Mars mission. Aviation Space and Environmental Medicine, 85(8), 793–797. 10.3357/ASEM.3907.2014 25199119

[bibr144-17470218221113935] Sultemeier D. R. Choy K. R. Schweizer F. E. Hoffman L. F. (2017). Spaceflight-induced synaptic modifications within hair cells of the mammalian utricle. Journal of Neurophysiology, 117(6), 2113–2124. 10.1152/JN.00240.2016 28228581 PMC5454470

[bibr145-17470218221113935] Tafforin C. (2013). The Mars-500 crew in daily life activities: An ethological study. Acta Astronautica, 91, 69–76. 10.1016/J.ACTAASTRO.2013.05.001

[bibr146-17470218221113935] Tanaka K. Nishimura N. Kawai Y. (2017). Adaptation to microgravity, deconditioning, and countermeasures. Journal of Physiological Sciences, 67(2), 271–281. 10.1007/s12576-016-0514-8 PMC10717636 28000175

[bibr147-17470218221113935] Thornton W. E. Bonato F. (2013). Space motion sickness and motion sickness: Symptoms and etiology. Aviation Space and Environmental Medicine, 84(7), 716–721. 10.3357/ASEM.3449.2013 23855067

[bibr148-17470218221113935] Thornton W. E. Moore T. P. Pool S. L. (1987). Fluid shifts in weightlessness. Aviation, Space, and Environmental Medicine, 58(9 Pt 2), A86–90. 3675511

[bibr149-17470218221113935] Ushakov I. B. Vladimirovich M. B. Bubeev Y. A. Gushin V. I. Vasil’eva G. Y. Gennad’evna Vinokhodova A. Shved D. M. (2014). Main findings of psychophysiological studies in the Mars 500 experiment. Herald of the Russian Academy of Sciences, 84(2), 106–114. 10.1134/S1019331614020063

[bibr150-17470218221113935] Van Ombergen A. Jillings S. Jeurissen B. Tomilovskaya E. Rumshiskaya A. Litvinova L. Nosikova I. Pechenkova E. Rukavishnikov I. Manko O. Danylichev S. Rühl R. M. Kozlovskaya I. B. Sunaert S. Parizel P. M. Sinitsyn V. Laureys S. Sijbers J. Eulenburg P. Z. Wuyts F. L . (2019). Brain ventricular volume changes induced by long-duration spaceflight. Proceedings of the National Academy of Sciences of the United States of America, 116(21), 10531–10536. 10.1073/pnas.1820354116 31061119 PMC6535034

[bibr151-17470218221113935] Van Ombergen A. Laureys S. Sunaert S. Tomilovskaya E. Parizel P. M. Wuyts F. L . (2017a). Spaceflight-induced neuroplasticity in humans as measured by MRI: What do we know so far? npj Microgravity, 3(1), 2. 28649624 10.1038/s41526-016-0010-8 PMC5445591

[bibr152-17470218221113935] Van Ombergen A. Wuyts F. L. Jeurissen B. Sijbers J. Vanhevel F. Jillings S. Parizel P. M. Sunaert S. Heyning P. H. V. D. Dousset V. Laureys S. Demertzi A . (2017b). Intrinsic functional connectivity reduces after first-time exposure to short-term gravitational alterations induced by parabolic flight. Scientific Reports, 7(1), 3061. 10.1038/s41598-017-03170-5 28607373 PMC5468234

[bibr153-17470218221113935] Wang Y. Zhou Y. Rao L.-L. L. Zheng R. Liang Z.-Y. Y. Chen X.-P. P. Tan C. Tian Z.-Q. Q. Wang C.-H. H. Bai Y.-Q. Q. Chen S.-G. G. Li S. (2017). Effect of 45-day −6° head-down bed rest on cooperation and aggression. Applied Cognitive Psychology, 31(5), 500–507. 10.1002/acp.3346

[bibr154-17470218221113935] Watenpaugh D. E. (2016). Analogs of microgravity: Head-down tilt and water immersion. Journal of Applied Physiology, 120(8), 904–914. 26869710 10.1152/japplphysiol.00986.2015

[bibr155-17470218221113935] Weber J. Javelle F. Klein T. Foitschik T. Crucian B. Schneider S. Abeln V. (2019). Neurophysiological, neuropsychological, and cognitive effects of 30 days of isolation. Experimental Brain Research, 237(6), 1563–1573. 10.1007/s00221-019-05531-0 30927043

[bibr156-17470218221113935] Werkhoven P. Snippe H. P. Alexander T. (1992). Visual processing of optic acceleration. Vision Research, 32(12), 2313–2329. 10.1016/0042-6989(92)90095-Z 1288008

[bibr157-17470218221113935] Yuan P. Koppelmans V. Reuter-Lorenz P. de Dios Y. Gadd N. Wood S. Riascos R. Kofman I. Bloomberg J. Mulavara A. Seidler R. (2018). Vestibular brain changes within 70 days of head down bed rest. Human Brain Mapping, 39(7), 2753–2763. 10.1002/hbm.24037 29528169 PMC6033666

[bibr158-17470218221113935] Yuan P. Koppelmans V. Reuter-Lorenz P. A. de Dios Y. E. Gadd N. E. Wood S. J. Riascos R. Kofman I. S. Bloomberg J. J. Mulavara A. P. Seidler R. D. (2016). Increased brain activation for dual tasking with 70-days head-down bed rest. Frontiers in Systems Neuroscience, 10, Article 71. 10.3389/fnsys.2016.00071 PMC4993791 27601982

[bibr159-17470218221113935] Zago M. Bosco G. Maffei V. Iosa M. Ivanenko Y. P. Lacquaniti F. (2004). Internal models of target motion: Expected dynamics overrides measured kinematics in timing manual interceptions. Journal of Neurophysiology, 91(4), 1620–1634. 10.1152/jn.00862.2003 14627663

[bibr160-17470218221113935] Zago M. Bosco G. Maffei V. Iosa M. Ivanenko Y. P. Lacquaniti F. (2005). Fast adaptation of the internal model of gravity for manual interceptions: Evidence for event-dependent learning. Journal of Neurophysiology, 93(2), 1055–1068. 10.1152/jn.00833.2004 15456796

[bibr161-17470218221113935] Zago M. Lacquaniti F. (2005a). Cognitive, perceptual and action-oriented representations of falling objects. Neuropsychologia, 43(2), 178–188. 10.1016/j.neuropsychologia.2004.11.005 15707903

[bibr162-17470218221113935] Zago M. Lacquaniti F. (2005b). Internal model of gravity for hand interception: Parametric adaptation to zero-gravity visual targets on earth. Journal of Neurophysiology, 94(2), 1346–1357. 10.1152/jn.00215.2005 15817649

[bibr163-17470218221113935] Zeng L.-L. Liao Y. Shen H. Liu X. Hu D. (2016a). Decoding brain states with simulated microgravity from baseline using functional connectivity of default network. In Wang R. Pan X. (Eds.), Advances in cognitive neurodynamics (V) (pp. 325–330). Springer. 10.1007/978-981-10-0207-6_45 PMC4805685 27066149

[bibr164-17470218221113935] Zeng L.-L. Liao Y. Zhou Z. Shen H. Liu Y. Liu X. Hu D. (2016b). Default network connectivity decodes brain states with simulated microgravity. Cognitive Neurodynamics, 10(2), 113–120. 27066149 10.1007/s11571-015-9359-8 PMC4805685

[bibr165-17470218221113935] Zhao X. Wang Y. Zhou R. Wang L. Tan C. (2011). The influence on individual working memory during 15 days -6° head-down bed rest. Acta Astronautica, 69(11–12), 969–974. 10.1016/j.actaastro.2011.07.003

[bibr166-17470218221113935] Zhou Y. Wang Y. Rao L.-L. Liang Z.-Y. Chen X.-P. Zheng D. Tan C. Tian Z.-Q. Wang C.-H. Bai Y.-Q. Chen S.-G. Li S. (2014). Disrutpted resting-state functional architecture of the brain after 45-day simulated microgravity. Frontiers in Behavioral Neuroscience, 8, Article 200. 10.3389/fnbeh.2014.00200 PMC4046318 24926242

[bibr167-17470218221113935] zu Eulenburg P. Caspers S. Roski C. Eickhoff S. B . (2012). Meta-analytical definition and functional connectivity of the human vestibular cortex. NeuroImage, 60(1), 162–169. 22209784 10.1016/j.neuroimage.2011.12.032

